# Solving the shepherding problem: heuristics for herding autonomous, interacting agents

**DOI:** 10.1098/rsif.2014.0719

**Published:** 2014-11-06

**Authors:** Daniel Strömbom, Richard P. Mann, Alan M. Wilson, Stephen Hailes, A. Jennifer Morton, David J. T. Sumpter, Andrew J. King

**Affiliations:** 1 Department of Mathematics, Uppsala University, Uppsala 75106, Sweden; 2 Structure and Motion Laboratory, The Royal Veterinary College, University of London, Hatfield, Hertfordshire AL9 7TA, UK; 3 Department of Computer Science, University College of London, Gower Street, London WC1E 6BT, UK; 4 Department of Physiology, Development and Neuroscience, University of Cambridge, Downing Street, Cambridge CB2 3DY, UK; 5 Department of Biosciences, College of Science, Swansea University, Swansea SA2 8PP, UK

**Keywords:** collective motion, agent-based model, sheep, sheepdog

## Abstract

Herding of sheep by dogs is a powerful example of one individual causing many unwilling individuals to move in the same direction. Similar phenomena are central to crowd control, cleaning the environment and other engineering problems. Despite single dogs solving this ‘shepherding problem’ every day, it remains unknown which algorithm they employ or whether a general algorithm exists for shepherding. Here, we demonstrate such an algorithm, based on adaptive switching between collecting the agents when they are too dispersed and driving them once they are aggregated. Our algorithm reproduces key features of empirical data collected from sheep–dog interactions and suggests new ways in which robots can be designed to influence movements of living and artificial agents.

## 1. Introduction

Determining how social organisms form and maintain swarm-like behaviour is a major scientific challenge that has been taken up by biologists, physicists, mathematicians and engineers [1–4]. Some of the most striking examples of this collective behaviour occur in the presence of threat; when flocks, shoals and herds aggregate and evade their predators [1]. A sheep flock's response to a herding dog is a classic example of what Hamilton [5] called the selfish herd theory, which posits that aggregations result from individual efforts to reduce their own predation risk by moving towards the centre of a group. Recent empirical evidence supports this sheep anecdote, showing that sheep show a strong attraction towards the centre of their flock with the approach of a sheepdog [6]. However, the fact that the flock tightens does not tell us how the dog is able to manoeuvre this aggregation and herd the flock towards a specific destination.

Many attempts have been made to gain an understanding of how a single agent can gather and herd a group of other agents [7–16]. With such knowledge comes numerous applications, for example in crowd control [17,18], cleaning up the environment [19], herding of livestock [20], keeping animals away from sensitive areas [21] and collecting/guiding groups of exploring robots [22]. Most research has adopted a theoretical approach, and sought to model the interaction of the agents based on attraction, repulsion and alignment models that are common in studies of collective animal behaviour [2,4,23–27]. One agent, the ‘shepherd’, is then given a different set of rules from the rest of the flock, which are repelled by the shepherd. In one class of models, the shepherd's rules prescribe a side-to-side movement behind the group while herding it towards the target [7,28]. Such algorithms are appropriate for herding small groups (see [16] for a review), but herding of larger groups (more than 40 individuals) typically requires multiple shepherds [28]. However, single sheep dogs can successfully herd flocks of 80 or more sheep both in their everyday work and in competitive herding trials [29,30]. So, what are the sheepdogs doing that the agent shepherds (or the flocking agents) are not?

Here, we propose a self-propelled particle model of local attraction–repulsion type to model herding of a group of interacting agents by one shepherd towards a predetermined destination. We begin by investigating how the success of the general algorithm depends on both group size and the degree of locality in the agent interactions. Then we focus on the shepherd dynamics that result from the application of the algorithm. Finally, we compare the model against empirical data obtained from real-life herding experiments with an Australian sheepdog and merino sheep [6].

## 2. Results

**Figure 1.**
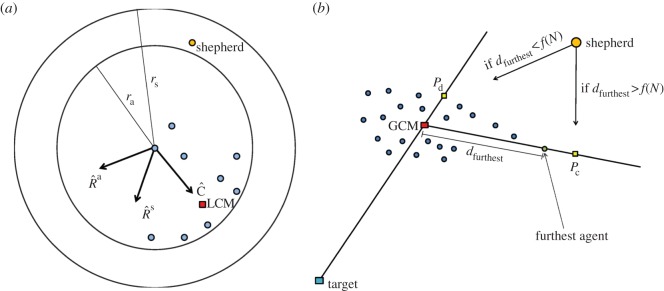
Interaction rules for the agents and the shepherd. (*a*) The agents are attracted to the LCM of their *n* nearest neighbours 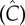, repelled from other agents within a distance of *r*_a_ 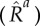 and repelled from the shepherd if it is within a distance of *r*_s_ 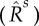. The new heading of the focal agent 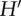 is a linear combination of the three vectors 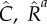 and 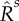, weighted by the corresponding model parameters *c*, *ρ*_a_, *ρ*_s_, plus a small inertia term 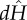 and a small noise term 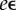. (*b*) In each time step, the shepherd does one out of three things depending on the position of the agents. If the shepherd is within 3*r*_a_ from any agent, its speed is set to zero. Otherwise, if all agents are within a distance *f*(*N*) from the GCM of the agents, then the shepherd aims towards the driving position *P*_d_ directly behind the flock relative to the target. Finally, if at least one sheep is further away than *f*(*N*) from the GCM, then the shepherd aims for the collecting position *P*_c_ directly behind the furthest away sheep relative to the GCM. (Online version in colour.)

The key features of our model are summarized below. A detailed account may be found in the Model and methods. Initially, *N* flocking agents are released at random positions in the upper right quarter of an *L* × *L* square field and a shepherd released outside this quarter. Each agent aims to stay away from the shepherd while remaining close to its *n* nearest neighbours. This behaviour of being attracted to nearby neighbours and repelled from potential threats is typical for sheep and many other herding animals [5]. Figure 1*a* illustrates the rules governing the agents. If an agent is further away than *r*_s_ from the shepherd, it remains stationary, except for occasional random movements. Regardless of distance to the shepherd, agents are repelled from other agents at very short distances of less than *r*_a_ and the unit vector 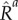 indicates the direction of this local repulsion. If an agent is within a distance *r*_s_ from the shepherd, the agent is attracted to the local centre of mass (LCM) of its *n* nearest neighbours, in the direction of the unit vector 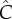, and at the same time repelled directly away from the shepherd in the direction of 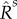. The new heading of the agent 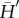 is then a linear combination of these three vectors weighted by corresponding model parameters *ρ*_a_, *c*, *ρ*_s_ plus a weak inertia term 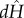 and a small noise term 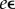. 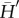 is then normalized and the agent moves a distance of *δ* in this direction. As only the direction, not the length, of 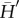 is important each weight gives the relative strength of the corresponding term. For example, *ρ*_a_ is the relative strength of repulsion from other agents, *c* is the relative strength of attraction to other agents and *ρ*_s_ is the relative strength of repulsion from the shepherd.

The shepherd's task is to collect and drive all agents down to the lower left corner of the field. In order to solve this task, we propose the following algorithm. If the shepherd is within 3*r*_a_ from any flocking agent, it does not move in this time step. Otherwise, the shepherd does one of two things depending on the position of the agents (figure 1*b*). If all agents are within a distance *f*(*N*) from their global centre of mass (GCM), then the shepherd aims towards the driving position *P*_d_ directly behind the flock relative to the target. We label this behaviour as ‘driving’. If at least one agent is further away than *f*(*N*) from the GCM, then the shepherd aims instead for the collecting position *P*_c_ directly behind this furthest away agent. We call this behaviour ‘collecting’. When collecting or driving the shepherd moves a distance of *δ*_s_.

**Figure 2.**
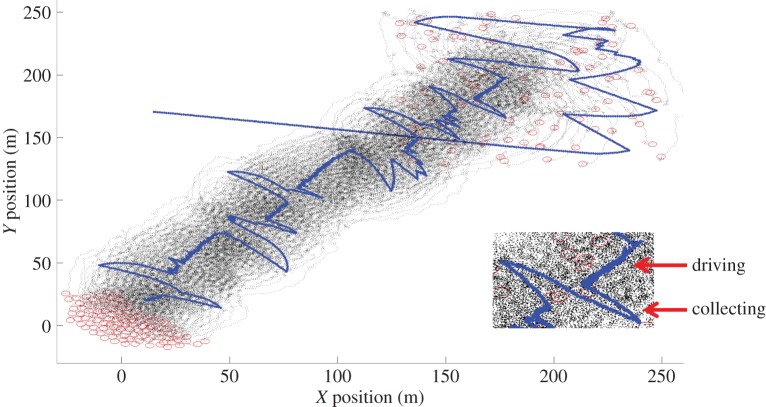
Simulation trajectories. The accumulated result of a simulation with 100 agents. The agents' starting and end positions are marked with circles and their trajectories throughout the simulation are thin lines. The trajectory of the shepherd (thick line) starts at (15,170) and goes directly for the agent furthest from the GCM at coordinates (245,140). When the shepherd approaches the agents aggregate and are eventually cohesive enough to start herding when the shepherd is at position (170,200). After a short straight driving phase, the shepherd is forced to go to one flank and then immediately the other to collect agents drifting off. This process of driving and collecting then goes on until the GCM of the group of agents is within 10 units from (0,0). The box highlights the driving phase and collecting phase which results in the side-to-side motion of the shepherd. (Online version in colour.)

Typical simulation results are shown in figure 2 and in the electronic supplementary material, video 1. Owing to agents being randomly placed at the upper right-hand quadrant of the *L* × *L* field, the shepherd tends to initially collect the agents until they are cohesive, at which point it starts to drive the group. Once the agents are mobile, the shepherd switches between driving and collecting modes until the task is completed and the agents are delivered to the target location in the lower left corner of the field. Visualizing the trajectories of the shepherd and agents throughout the simulations, we observe a side-to-side motion of the shepherd behind the agents (figure 2). This motion is not explicitly coded in our shepherd's behaviours. Instead, it emerges as a consequence of the shepherd switching between driving and collecting.

**Figure 3.**
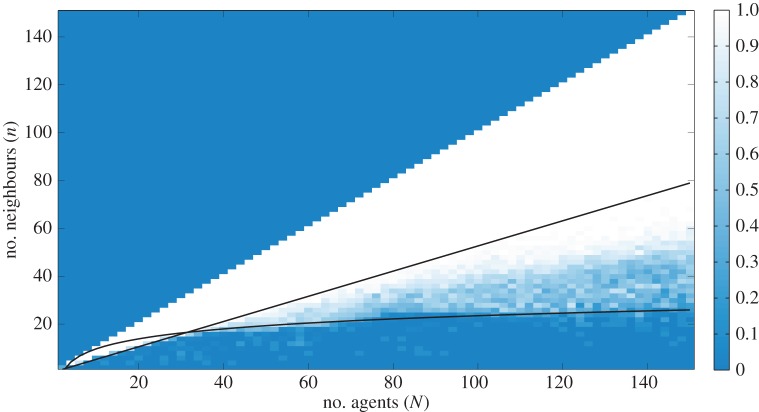
Proportion of successful shepherding events within 8000 time steps as a function of 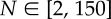 and nearest neighbours 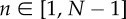. We see that in the global case *n* = *N* − 1 and down to roughly *n* = 0.53*N* the algorithm is always successful. For *N* > 30, there is a transition region below the line *n* = 0.53*N* and above *n* ≈ 3log(*N*) where the probability of success drops from 1 to rare at a rate that is decreasing with *N*. Finally, in the region under the curve min(0.53*N*, 3log(*N*)) success is rare and sporadic. The other parameters are the typical values listed in table 1 except for *r*_s_ = 45. (Online version in colour.)

To evaluate the performance of the shepherding algorithm, we investigate how often it completes the task successfully within 8000 time steps for groups of 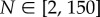 agents. In particular, we explore how the number of nearest neighbours 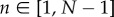 affects the performance of the algorithm. Figure 3 shows the proportion of successful shepherding events as a function of *N* and *n* over 50 simulations. In the global case *n* = *N* − 1 and down to *n* = 0.53*N*, the algorithm is always successful. For *N* > 30, there is a transition region below the line *n* = 0.53*N* and above *n* ≈ 3log(*N*) where the probability of success drops from 1 to rare. In the region under the curve min(0.53*N*, 3log(*N*)) success is very rare.

We tested whether our model matched data collected using a back-mounted global positioning system (GPS) attached to *N* = 46 sheep and a working farm dog [6,31]. Electronic supplementary material, video 2, shows the data collected in the three experiments. For each herding event, the following can be observed: first, the dog approaches the sheep, and the sheep aggregate. The dog then positions itself behind the flock relative to the end position and starts driving it forward. As the flock is driven forward, individuals at one or both flanks begin to drift away from the overall flock centre of mass. The dog corrects for this by approaching the flank sheep and positions itself behind the sheep relative to the centre of the flock. The dog then returns to herding the flock towards the target.

**Figure 4.**
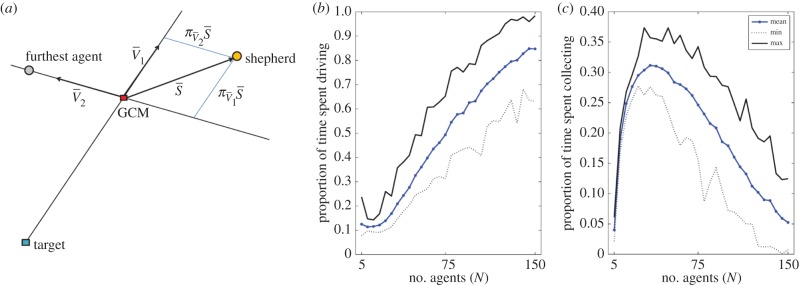
Projections used to define the driving and collecting modes and how the proportion of time spent driving and collecting depends on the number of agents. (*a*) How the centroid vector *v*_1_, the furthest agent vector *v*_2_ and shepherd vector *S* are set up. Three measurements related to these vectors will be used in comparing the model to the sheep data (figure 5). The projection of *S* on *v*_1_ denoted by 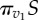, the projection of *S* on *v*_2_ denoted by 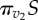 and the length of *S*. (*b*) Proportion of time the shepherd spends driving 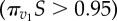 as a function of group size (*N*) in the global case (*n* = *N* − 1) over 100 simulations. (*c*) Proportion of time spent collecting 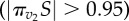 as a function of group size (*N*) in the global case over 100 simulations. The other parameters are the typical values listed in table 1. (Online version in colour.)

To quantitatively compare the model with the experimental data, we calculate the projections of the shepherd vector 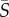 onto the centroid vector *v*_1_ and the furthest agent vector 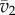, respectively (figure 4*a*). These projections 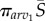 and 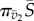 provide us with information about where the shepherd is relative to the centre of mass of the flock. If 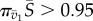, the shepherd is directly behind the flock relative to target. If 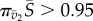, the shepherd is on the same side of the flock as the furthest agent and if 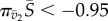, it is on the opposite side. We calculate these projections in each time step of the simulation and in the data and present distributions showing the proportion of time steps the shepherd spent in a certain position. These measures capture both the driving mode, where the shepherd is directly behind or in front of the flock relative to the target (i.e. 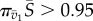), and the collecting mode when the shepherd is directly behind, or on the opposite side of the flock, relative to the furthest agent (i.e. 
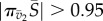
). Figure 4*b*,*c* shows how the proportion of time driving and collecting depends on the number of agents in simulations in the global case. The time the shepherd spends collecting agents increases until *N* = 40, and then decreases linearly (figure 4*c*), with a corresponding increase in time spent driving (figure 4*b*) as *N* increases and the flock is less likely to fission.

**Figure 5.**
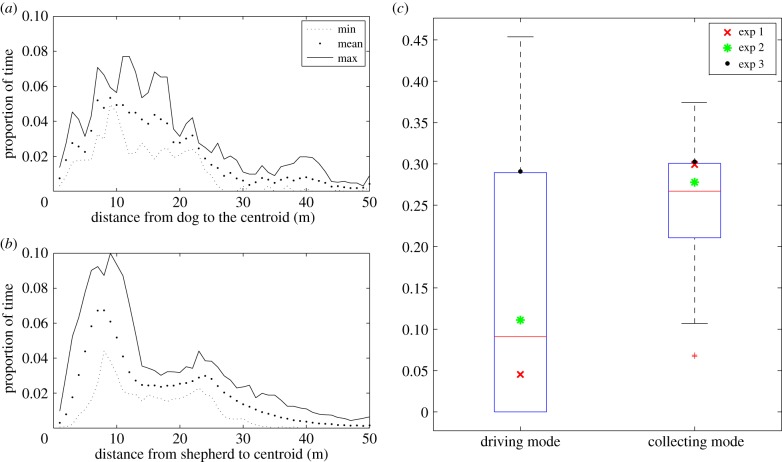
Comparison of the model with data for 46 sheep. (*a*) The proportion of time the dog spent at a certain distance from the GCM of the sheep (length of vector *S* in figure 4) over the three trials. (*b*) The proportion of time the shepherd spent at a certain distance from the GCM of the agents over 100 simulations. The overall shape of the distance distributions in experiments and simulations agree and in particular both exhibit a peak at around 10 m. (*c*) The proportion of time the dog/shepherd spent in driving mode (directly behind the flock relative to the target) and in collecting mode (on the same or opposite side of the flock as the furthest sheep). The boxplots illustrate the proportion of time the shepherd spent in driving or collecting mode over 100 simulations and the points represent the proportion of time the dog spent driving or collecting during three experimental trials. The parameters used in the simulation are the typical values listed in table 1 except for *n* = 46, *r*_s_ = 75 m, *r*_a_ = 1 and *e* = 0.1. The experimental data can be seen in the electronic supplementary material, video 2, and simulations with these parameter values in video 3. (Online version in colour.)

Figure 5*a* shows the minimum, mean and maximum distance between the shepherd and the centre of mass of the agents throughout the three trials. We compare this with the result of simulations in which the parameters are set to mimic the behaviour of real sheep and sheepdogs (figure 5*b*; electronic supplementary material, video 3). The overall shapes of the distributions match and both include the peak at around 10 m. Figure 5*c* shows that the proportion of time the dog spends in the driving and collecting modes is consistent with the shepherd behaviour in simulations. The box plot shows the result of 100 simulations and the results of each of the three trials are marked with dots. The full distributions of the centroid and furthest sheep projection values in simulations and in the data are presented in the electronic supplementary material, figure S1. We see that the projection on the centroid vector 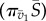 is peaked at 1, indicating a position behind the flock (electronic supplementary material, figure S1*a*,*c*). The distribution of the projection on the furthest sheep vector 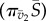 has a bimodal structure, peaking at −1 and 1, which indicates a position either on the side of the flock with the furthest sheep, or on the opposite side of the flock (electronic supplementary material, figure S1*b*,*d*). This results from both the dog's positioning itself to collect the furthest sheep, and the resulting attempted ‘escape’ of sheep on the other side of the flock. We also investigated how the distance from the initial release site to the target affected success rate and time to completion. In each simulation, 46 agents were initially positioned randomly within a 50 × 50 square centred at the point 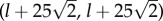. We performed 100 simulations for each value of distance to target *l*, with *l* increasing from 10 to 500, and the time to completion was measured. The result is shown in the electronic supplementary material, figure S2, and we see that the success rate is unaffected and that the average time to completion increases linearly with distance to target.

**Figure 6.**
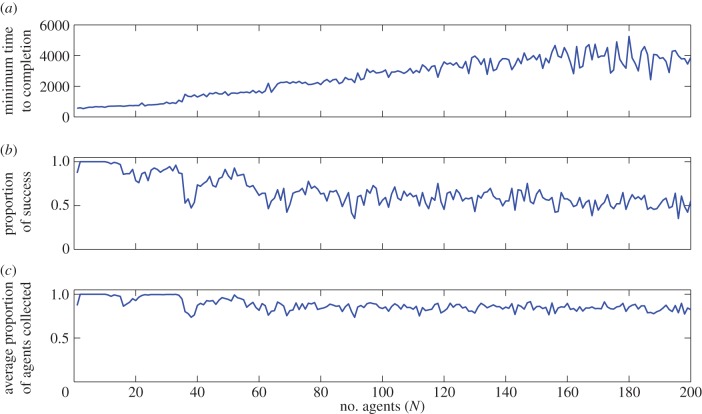
Performance of the local shepherd as a function of number of agents (*N*) from 1 to 200 over 100 simulations. (*a*) The minimum time to completion as a function of number of agents. (*b*) The proportion of success. (*c*) The average proportion of agents collected as a function of number of agents. The number of nearest neighbours was *n* = 20 and the other parameters as listed in table 1 except *L* = 300, *t*_max_ = 40 000, *r*_a_ = 3 and *h* = 0.3. (Online version in colour.)

Although our model is consistent with the data and allows herding of the 46 sheep used in the experiment, our algorithm is not guaranteed to succeed when *n* < *N*/2 and often fails when 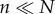. One way of overcoming this problem and potentially allow the shepherd to deal with groups of arbitrary size is to programme it to sequentially bring in subgroups of a size it can handle. To test this idea, we implemented a shepherd that employs the algorithm on the LCM of the *n*_s_ nearest neighbours rather than the GCM of all agents. Initial investigations showed that the shepherd can get stuck in the centre of mass of several symmetrically distributed subgroups and is thereby unable to complete the task. To counteract this problem, we also introduced a blind zone behind the dog specified by an angle *β* [32] (see the Models and methods for details). This modification improved the situation. In 56 out of 100 simulation runs a shepherd acting on its *n*_s_ = 20 nearest neighbours successfully brought in groups of *N* = 201 agents with *n* = 20. Electronic supplementary material, video 4, shows six such simulations. In the first three simulations, the shepherd is successful, often bringing in groups of more than *n*_s_ = 20 agents at a time. However, the last three simulations show typical situations where the shepherd gets stuck and fails to complete the task. In these runs, the shepherd is typically trapped between two or more clusters. Three quantitative measures of the local shepherd's performance as a function of number of agents (*N*) are presented in figure 6. For each *N*, we ran 100 simulations and calculated the minimum time to completion (figure 6*a*), proportion of successful herding events (figure 6*b*) and finally the average proportion of agents collected over the 100 simulations (figure 6*c*). Minimum time to completion suggests that in the optimal case, the time to completion increases approximately linearly with number of agents (minimum completion time = 20*N* + 630). The proportion of successful trials decreases from 1 for small number of agents down to approximately 0.5 for *N* = 200. However, even in the cases where the shepherd ultimately fails, it tends to bring in a majority of agents before. The average number of agents collected decreases with number of agents, but slowly, and even in simulations with 200 agents the shepherd manages to bring in 80% of the total number of agents over 100 simulations.

## 3. Discussion

The model we have presented offers a solution to the shepherding problem. Using a set of simple heuristics, we show that a shepherd can herd autonomous, interacting agents towards a target destination. The shepherd heuristics are based on adaptive switching between collecting agents when they are too dispersed and driving them once they are aggregated. These rules function to (i) reduce the probability that the group splits and (ii) allow the shepherd to keep the group moving towards a target location. A side-to-side motion of the shepherd also emerges as a consequence of these rules, a feature which has previously been hard-coded into shepherd movement rules in other models to improve efficiency [7,16,28].

There are a number of aspects of both the shepherd and the agent behaviour in our model that are consistent with real herding events involving sheep and a sheepdog (figure 5). Instead of weaving side-to-side behind a flock at some frequency, our shepherding algorithm results in the shepherd driving the group when it is cohesive and actively seeking and collecting those that drift out at the edges. This is exactly the type of behaviour that we see in our sheep–sheepdog datasets. The visual similarities between the model and the data (electronic supplementary material, videos 2 and 3) are corroborated by quantitative comparisons of the data and our model (figure 5).

The plausibility of our model relies upon two assumptions. The first is that the dog can estimate the space between the sheep, irrespective of their metric distance. This seems reasonable given the border collie, a classic sheepdog breed, is said to use a direct stare to herd the flock [29], and similar heuristics-based models have proved useful in understanding the behaviour of pedestrians in crowds [33]. Nevertheless, it would be useful to gather further evidence using, for example, eye-tracking systems to determine shifts in the dog's visual attention. Also, in our field experiments, we used an experienced dog which was given minimal direction. It would be interesting to conduct experiments with multiple dogs and owners of dogs of varied abilities; this way, we would be begin to investigate the role of task familiarity and learning in herding performance. Our second assumption is that agents are attracted to the centre of mass of at least half of the total number of agents (figure 3). At larger group sizes, this means that agents interact with many neighbours, which is at odds with other theoretical models of flocking in which agents tend to interact locally with a small number of individuals [16]. It is nonetheless consistent with a sheep flock's initial responses to the approach of a herding sheepdog [6], and with empirical data on bird flocking in which a topological interaction is required to maintain flock cohesion under perturbations [34].

Although our algorithm is consistent with the data, and unlike previous models [16] allows herding of large groups, it is not guaranteed to succeed when agents interact with less than half of the total group size. Under these conditions, the group of agents is likely to split into two or more stable subgroups. As our intention was to create a model that was not only applicable to the sheep–sheepdog scenario, but also to similar phenomena such as cleaning the environment, we augmented our basic shepherding algorithm with a mechanism to allow the shepherd to detect that the group has split and then bring each subgroup in sequentially. To do this, we employed the same shepherding algorithm on the LCM of nearby agents, rather than on the GCM (note that this might also be applicable to the sheepdog at large group sizes, because it would not be able to see the entire flock, but we do not have data on this). Merging of separated agents has been discussed in [8] but the approach of splitting the task into one for each subgroup is, as far as we know, new. However, at present this extended local shepherd algorithm is not always successful, rather it has a success rate of about 80% as a result of the shepherd getting stuck between collecting two subgroups of agents. In practice (e.g. with a herding dog or herding robot), an instruction could be given which could rectify this.

Our approach should support efficient designs for herding autonomous, interacting agents in a variety of contexts. Obvious cases are robot-assisted herding of livestock [20], and keeping animals away from sensitive areas [21], but applications range from control of flocking robots to cleaning up of environments and human crowd control. In the case of flocks of mobile robots, for example, engineers have designed virtual or explicit leaders to guide groups to target headings, or else assumed that a heading is sensed by the whole group [22]. A simpler alternative is to shepherd such groups, using the algorithm which we have described here. This would be particularly useful for guiding robots back to a base after completion of some task. In the case of cleaning up of environments, multi-robot systems have been proposed to help clean up marine oil spills, and specifically prevent spills from spreading wider [35]. It would be fascinating to explore how our algorithms performed in this task and in other scenarios where fluids or granular media need collecting/corralling. The algorithm may also be applied to situations where crowds of people have little information and there is a tendency to imitate the behaviour of each other. This is especially common where visibility is poor, and people need to escape from a smoky room [36]. In such situations, it may be possible to herd the movements of people to exits using a shepherd robot.

Finally, returning to biology, our results also inform our understanding of animal collective behaviour in the presence of threat [37]. It is tempting to envisage a similar set of rules to those we describe for our shepherd guiding the behaviour of predators attacking flocking prey. While the same ability to estimate space between prey might be important to understanding and building a mechanistic understanding of predator movement rules, these rules will not be the same as those used here. The goal of the shepherd (and our sheepdog example) is to keep the flock together and manoeuvre it as a single unit; a predator's goal is typically the opposite, namely to break up aggregations and isolate individuals as potential targets [38].

## 4. Model and methods

### 4.1. Model

Initially, *N* agents are randomly positioned in the upper right quarter of an *L* × *L* square and a shepherd released in the lower left quarter. The square is not enclosed so the agents and shepherd may leave it at a later time. Denote the position of the shepherd by 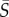 and the position of the *i*th agent by 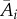. If an agent is further away than *r*_s_ from the shepherd it grazes. That is, it is typically stationary, but exhibits small random movements. If the distance to the shepherd is shorter than *r*_s_, then each agent *i* will be repelled directly away from it in the direction of 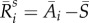 and at the same time will be attracted to the centre of mass of its *n* nearest neighbours 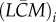 in the direction of 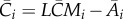. Agents are also locally repelled from each other, so that if two or more agents are within a distance of *r*_a_ of each other there will be a repulsive force acting to separate them. More precisely, if agent *i* has *k* neighbours within a distance of *r*_a_ at positions 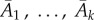, the repulsive force on *i* is defined by4.1
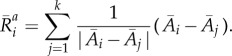
The heading agent *i* will take in the next time step 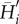 is a linear combination of these forces (normalized) plus inertia 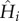 and an error term 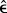 (see figure 1), which can be described as follows:4.2
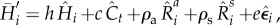
where the weights are chosen so that *ρ*_a_ > *c* > *ρ*_s_ > *h*. The reasons for this inequality are that agent-to-agent repulsion *ρ*_a_ must be dominating in order for any group size to be maintained and that in the real world sheep tend to aggregate rather than immediately disperse in the presence of a dog [6]. Therefore, we assume that local attraction between agents is stronger than repulsion from the shepherd *c* > *ρ*_s_. Finally, the tendency to proceed in the previous direction *h* is included to prevent sharp turns and smoothen trajectories and should be subordinate to all interactions. The typical values for these parameters in simulations are *ρ*_a_ = 2, *c* = 1.05, *ρ*_s_ = 1 and *h* = 0.5. Agent *i* will then move a distance of *δ* in this direction 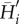 and its new position is given by4.3
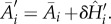
See table 1 for an overview of the parameters of the model.

**Table 1.** The parameters of the model. Notation, description and typical values used in simulations.

parameter	description	typical values
*L*	side length of initial square field	150 m
*agent parameters*		
*N*	total number of agents	1 − 201
*n*	number of nearest neighbours	1 − 200
*r*_s_	shepherd detection distance	65 m
*r*_a_	agent to agent interaction distance	2 m
*ρ*_a_	relative strength of repulsion from other agents	2
*c*	relative strength of attraction to the *n* nearest neighbours	1.05
*ρ*_s_	relative strength of repulsion from the shepherd	1
*h*	relative strength of proceeding in the previous direction	0.5
*e*	relative strength of angular noise	0.3
*δ*	agent displacement per time step	1 m ts^−1^
*p*	probability of moving per time step while grazing	0.05
*shepherd parameters*		
*δ*_s_	shepherd displacement per time step	1.5 m ts^−1^
*P*_d_	driving position	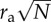 m behind the flock
*P*_c_	collecting position	*r*_a_ m behind the furthest agent
*e*	relative strength of angular noise	0.3
*for local shepherd*		
*n*_s_	number of nearest agents the local shepherd operates on	20
*β*	blind angle behind the shepherd	*π*/2

The shepherd's task is to collect all agents into one flock and herd them to the lower left corner of the *L* × *L* square. When the centre of mass of the flock (GCM) is within a certain distance from the origin, the shepherding task is completed. While shepherding the shepherd decides on one of two possible moves, collect or herd, at each time step, which depends on whether all agents are within a distance of *f*(*N*) from the GCM (see figure 2). 
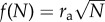
 would require the flock to be perfectly circular and so to allow for asymmetry of the flock, we take *f*(*N*) = *r*_a_*N*^2/3^. If all agents are not within *f*(*N*), the shepherd moves towards the point *P*_c_ to collect the agent furthest from the GCM at position *A*_f_. If the flock is cohesive, that is, all agents are within *f*(*N*), the shepherd positions itself at *P*_d_ to drive the flock. The shepherd attempts to go in a straight line towards these points but if it gets within 3*r*_a_ of an agent, its speed *δ*_s_ is set to 0. 3*r*_a_ was selected because of our observations of our sheepdog in the field, where the dog would rarely approach the flock at close range (since this causes the flock to fission). The shepherd experiences the same noise as the agents 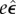; this noise is critical for resolving dead-lock situations. The shepherd will repeat this until the GCM is within a certain distance from the origin.

### 4.2. Local shepherd

The task of the local shepherd is to complete the original task of delivering all *N* agents down to the lower left corner by sequentially bringing in subgroups of a size it can handle. It starts by going to pick up the first subgroup, which it brings in. The shepherd repeats this process until all agents are brought in. We denote the number of agents that have not been brought in yet by *N_t_*. The agents behave exactly like in the original model but the shepherd now employs the algorithm on the LCM of the min(*N_t_*, *n*_s_) nearest agents within visual range (*LCM_t_*) instead of the GCM of all agents. The visual range of the shepherd is limited by the inclusion of a blind zone behind it relative to the detected *LCM_t_* specified by the angle *β*. The reason for including this blind zone was to overcome the problem of the shepherd being encircled by subgroups of agents and from then on unable to move. Electronic supplementary material, video 4, shows both successful and unsuccessful trials with a local shepherd using *n*_s_ = 20 and *N* = 201 agents with *n* = 20 and the other parameters as listed in table 1 except *L* = 300, *t*_max_ = 40 000, *r*_a_ = 3 and *h* = 0.3. The movie was constructed by recording every 100th frame of the simulations.

### 4.3. Sheep flock and herding dog

A flock of 46 female merino sheep (*Ovis aries*) aged 3 years and with a mean ± s.e.m. body weight of 52 ± 6 kg was used. Throughout the experiments, the sheep were housed in a 5 ha field and given ad libitum access to hay and water on all days. A trained female Australian Kelpie working farm dog was used to herd the sheep. All trials were undertaken in South Australia in March 2010. For each trial, the dog was directed verbally to herd the flock to the gate of the field, with minimal guidance (given the command ‘bring them home’). One herding event was recorded per day.

### 4.4. Sheep and dog movement data

All sheep and the sheepdog were fitted with a ‘data-logger’ during all herding events. The loggers are an in-house design and comprise a GPS module capable of recording single frequency L1 raw range data at 10 Hz (uBlox LEA-4T GPS module), a GPS patch antenna, MSP430 microcontroller and a rechargeable 2200 mAh lithium polymer battery. The logger was set to record raw pseudo-range GPS data at 1 Hz, which were saved to a micro-SD card. These components were mounted and housed in a sealable plastic box and attached to a standard sheep harness (Rurtec, Hamilton, New Zealand), or dog harness purchased from a local store. The logger and harness had a total mass of 530 g (150 g data logger, 381 g harness), which was 1% of mean sheep body mass, and has been shown not significantly to alter key locomotion parameters of sheep within this managed population [6]. A Novatel FlexPak G2L/OEM4 GPS base station was also mounted with a clear sky view on top of a grain silo at the location (approx. 6 m above ground level) providing synchronized measurements that were used to improve accuracy in post-processing. GPS data for loggers and base station were post-processed in differential mode using Waypoint Graf-Nav v. 8.10 (www.novatel.com). This approach allows carrier phase ambiguity resolution/a fixed integer kinematic solution and an absolute positional accuracy of 10–20 cm. Much of the error in positional accuracy was consistent across loggers and Gaussian in nature. For further details on post-processing, see [6,31]. Data were then analysed using Matlab v. R2010.

## Supplementary Material

Figure S1

## Supplementary Material

Figure S2
