# Supplementary figures and images for: Solving the shepherding problem: heuristics for herding autonomous, interacting agents
Source: J R Soc Interface. 2014 Nov 6;11(100):20140719. doi: 10.1098/rsif.2014.0719 (PMC4191104; doi:10.1098/rsif.2014.0719)

a)

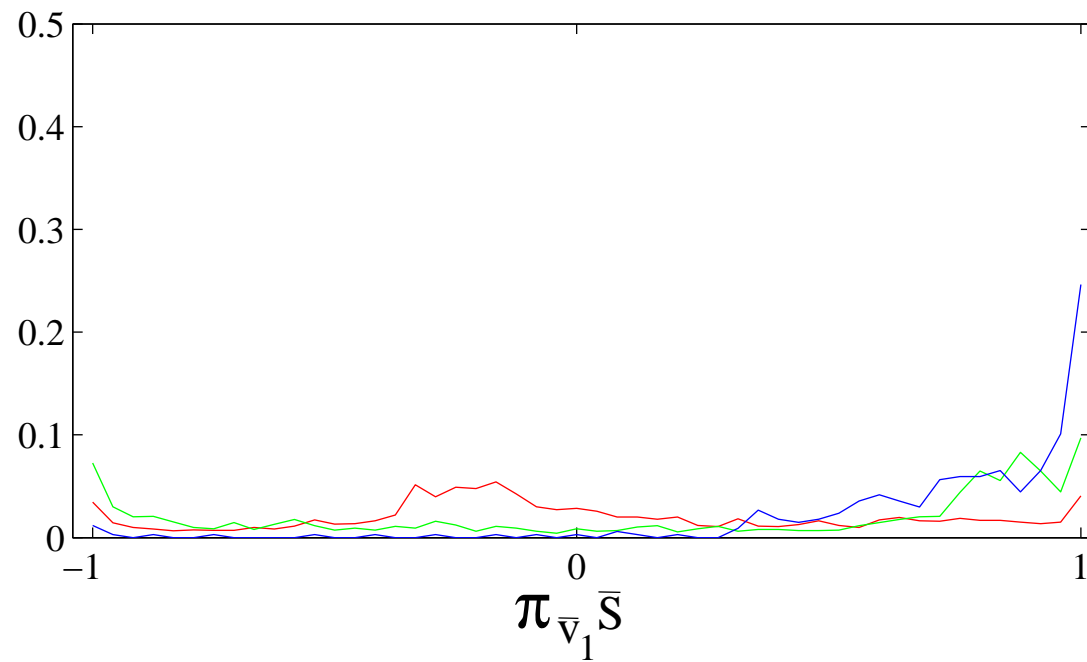

b)

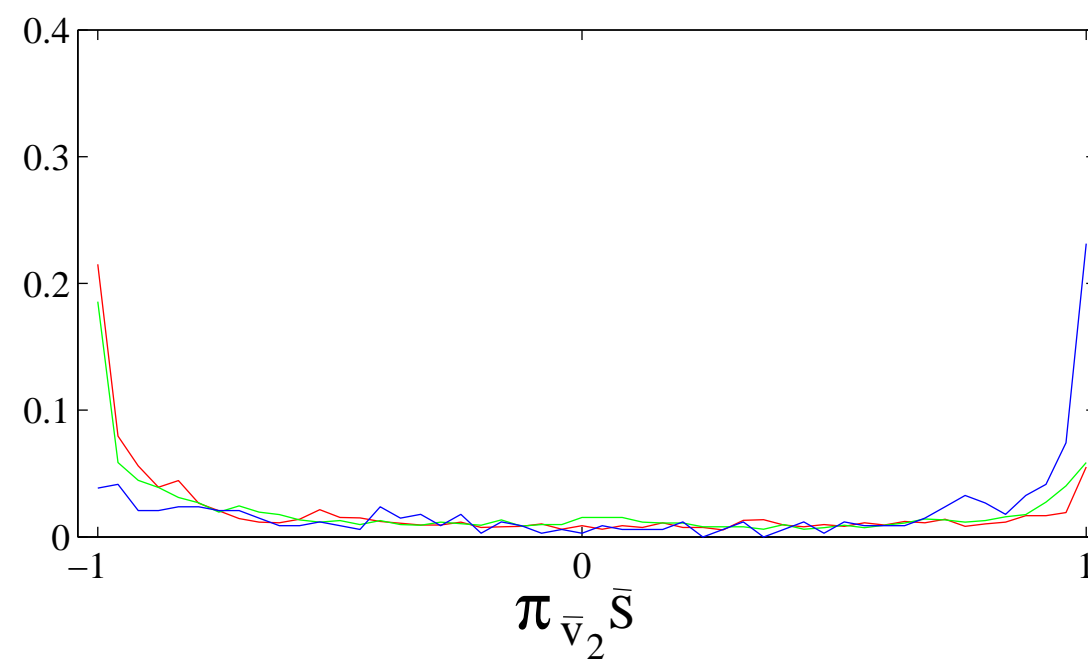

c)

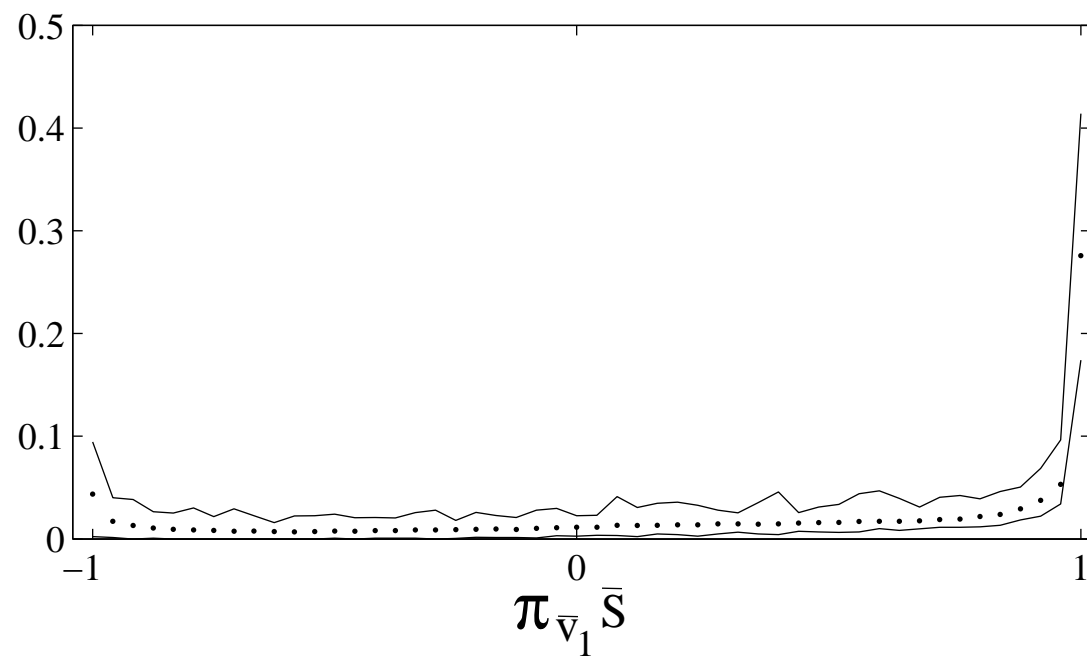

d)

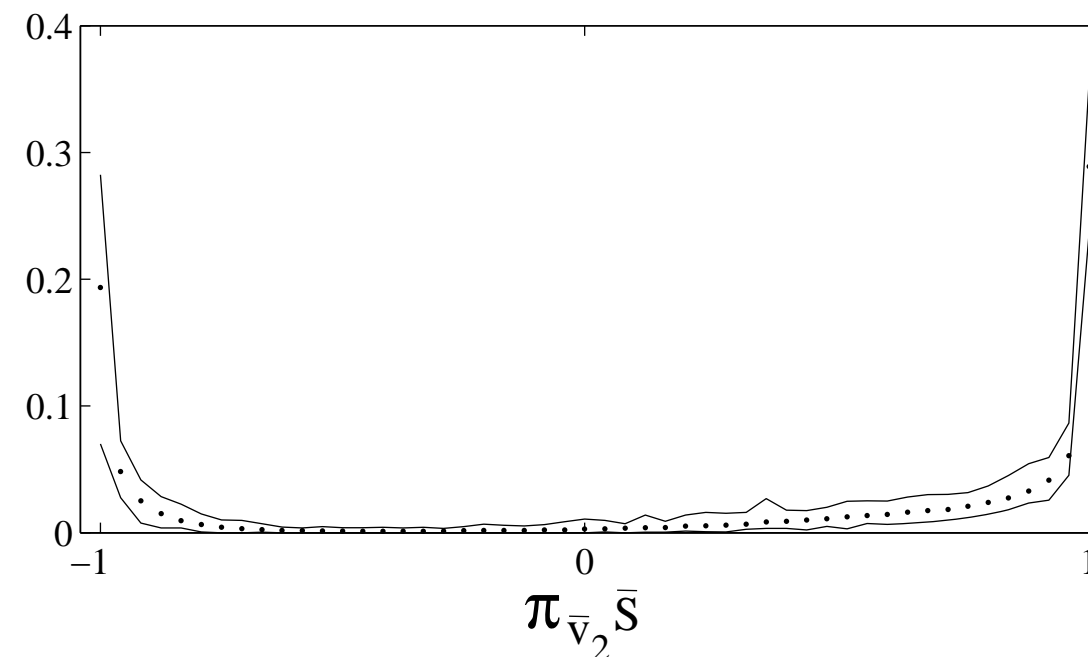

Supplement: Figure S1 [file rsif20140719supp1.pdf]

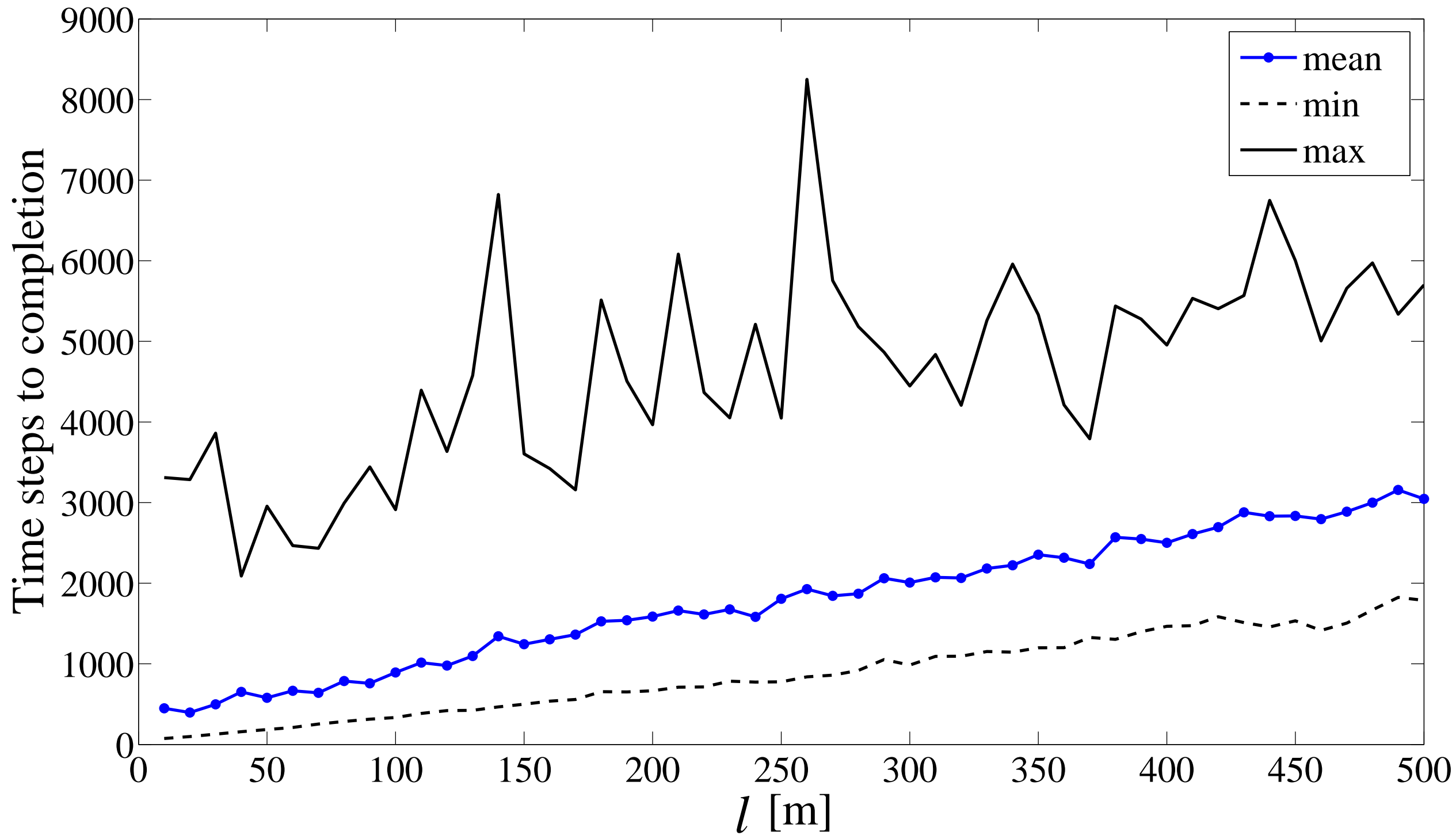

Supplement: Figure S2 [file rsif20140719supp2.pdf]
